# Cardiac impairments in postacute COVID‐19 with sustained symptoms: A review of the literature and proof of concept

**DOI:** 10.14814/phy2.15430

**Published:** 2022-08-22

**Authors:** Jyotpal Singh, Lanishen Bhagaloo, Eric Sy, Andrea J. Lavoie, Payam Dehghani, Holly A. Bardutz, Cameron S. Mang, Josef Buttigieg, J. Patrick Neary

**Affiliations:** ^1^ Faculty of Kinesiology and Health Studies University of Regina Regina Saskatchewan Canada; ^2^ Department of Cardiology Prairie Vascular Research Inc, Saskatchewan Health Authority Regina Saskatchewan Canada; ^3^ Gateway Alliance Medical Regina Saskatchewan Canada; ^4^ College of Medicine University of Saskatchewan Saskatoon Saskatchewan Canada; ^5^ Department of Family Medicine Saskatchewan Health Authority Regina Saskatchewan Canada; ^6^ Department of Critical Care Saskatchewan Health Authority Regina Saskatchewan Canada; ^7^ College of Medicine University of Saskatchewan Regina Saskatchewan Canada; ^8^ College of Graduate and Postdoctoral Studies University of Saskatchewan Saskatoon Saskatchewan Canada; ^9^ Faculty of Science, Department of Biology University of Regina Regina Saskatchewan Canada

**Keywords:** diastolic, echocardiography, global longitudinal strain, long COVID, right ventricle

## Abstract

Although acute COVID‐19 is known to cause cardiac damage in some cases, there is still much to learn about the duration and relative permanence of the damage that may occur. Long COVID is a condition that can occur when COVID‐19 symptoms remain in the postviral acute period. Varying accounts of long COVID have been described across the literature, however, cardiac impairments are sustained in many individuals and cardiovascular assessment is now considered to be an expected follow‐up examination. The purpose of this review and proof of concept is to summarize the current research related to the assessment of cardiac function, including echocardiography and blood biomarker data, during the follow‐up period in patients who recovered from COVID‐19. Following a literature review, it was found that right ventricular dysfunction along with global longitudinal strain and diastolic dysfunction are common findings. Finally, more severe acute myocardial injury during the index hospitalization appears to exacerbate cardiac function. The available literature implies that cardiac function must be monitored in patients recovered from COVID‐19 who remain symptomatic and that the impairments and severity vary from person‐to‐person. The proof‐of‐concept analysis of patients with cardiac disease and respiratory disease in comparison to those with sustained symptoms from COVID‐19 suggests elevated systolic time interval in those with sustained symptoms from COVID‐19, thus reducing heart performance indices. Future research must consider the details of cardiac complications during the acute infection period and relate this to the cardiac function in patients with long COVID during mid‐ and long‐term follow‐up.

## Graphical abstract

Cardiac parameters are shown to be impaired in follow‐up research on patients recovered from COVID‐19. Sustained symptoms are not always present with the associated cardiac impairments.

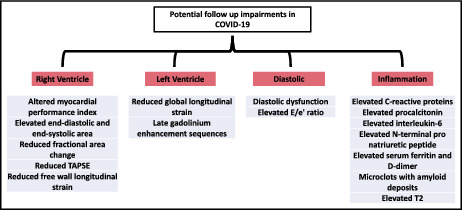

## 1 INTRODUCTION

Coronavirus disease‐2019 (COVID‐19) can result in long‐lasting cardiac dysfunction. Whether there are direct correlations with symptoms in this population is not known. “Long COVID” or postacute COVID‐19 syndrome (PACS) is difficult to define syndrome in which patients display multi‐organ complications including cardiac, respiratory, hematology, and autonomic nervous system dysregulation several weeks after first contracting COVID‐19. Functional problems following apparent recovery from COVID‐19 have also been reported, including chest pain, fatigue, dyspnea, tinnitus, and memory/cognitive impairment, among other issues (Guedj et al., 2021; Liguori et al., 2020). However, there is extreme heterogeneity in the clinical presentation of the patients, which makes this condition problematic to define. The purpose of this review is to summarize the available literature pertaining to cardiac sequelae in both patients with PACS/long COVID and those completing follow‐up assessments. We focus on systolic and diastolic (dys)function, right ventricular (RV) function, and inflammation and discuss potential mechanisms for long‐term cardiac symptoms. The studies observing changes in cardiac function were found by using a combination of the keywords “Cardiac” and “Long COVID” and “Postacute COVID‐19” and “echocardiography” and “cardiac cycle timing intervals” in PubMed and MEDLINE, with references to articles, also being included.

### 1.1 Definition

Long COVID or PACS can be broadly considered as a condition displaying “Prolonged multi‐organ symptoms and complications beyond the initial period of acute infection and illness” (Venkatesan, 2021). A UK survey showed that across positive cases, approximately 20% of individuals exhibit symptoms for a period of 5 weeks or longer and 10% for 12 weeks or longer (Statistics. OfN, 2020). These statistics led the National Institute for Health and Care Excellence (NICE), Scottish Intercollegiate Guidelines Network, and the Royal College of General Practitioners to provide guidelines for patients recovered from COVID‐19 with sustained symptoms. Collectively, they provided two definitions: post‐acute COVID‐19, defined as an “ongoing symptomatic COVID‐19” for people who still have symptoms between 4 and 12 weeks after the start of acute symptoms and “post‐COVID‐19 syndrome” for people who still have symptoms more than 12 weeks after the start of acute symptoms (Venkatesan, 2021).

### 1.2 Timeline and symptoms

From the available research, symptoms in patients with PACS predominantly include chest pain and palpitations,(Satterfield et al., 2021) with chest pain found in up to 21% and 5% of survivors at 60 days (Carfi et al., 2020) and 6 months(Huang et al., 2021), respectively. In comparison, palpitations appear to be persistent in up to 9% of survivors at the 6‐month follow‐up (Huang et al., 2021). Data obtained by the US Centers for Disease Control and Prevention (CDC) through a telephone survey demonstrated that 35% of symptomatic adults who had a positive outpatient test result for SARS‐CoV‐2 infection had continued to experience symptoms 2–3 weeks after testing, with 65% returning to normal within 5–12 days (Tenforde et al., 2020). Even in those 18–34 years of age with no chronic medical conditions, 1 in 5 was not back to their usual state of health within the 2–3‐week follow‐up (Tenforde et al., 2020).

## 2 CARDIAC FUNCTION

### 2.1 Left ventricular (LV) function

Acute COVID‐19 can cause myocardial injury and impact systolic function in severe cases, with parameters such as left ventricular ejection fraction (LVEF) associated with in‐hospital mortality (Silverio et al., 2021). Two‐dimensional speckle tracking echocardiography approximately 30 days post‐COVID‐19 treatment showed abnormal global longitudinal strain in 28 of 74 patients, implying subclinical myocardial deformation (Ozer, Candan, et al., 2021). These patients did not have any history of cardiac comorbidities which can alter LV function (Ozer, Candan, et al., 2021). Global longitudinal strain is suggestive of LV function and is a more accurate assessment of LV in comparison to LVEF in acute coronary syndrome (ACS) patients (Karlsen et al., 2019). Global longitudinal strain impairments can be sustained even at a 2‐month follow‐up period (Lassen et al., 2021). In some cases, 6‐month follow‐ups have shown that cardiac damage during the index hospitalization can result in a greater risk of prolonged cardiac damage, including late gadolinium enhancement sequences which can imply cardiac fibrosis (Wu et al., 2021). These patients did not have a history of coronary heart disease or cardiomyopathy (Wu et al., 2021). However, research is also available to show that LVEF is normal even in patients suffering prolonged symptoms after acute infection, such as dyspnea on exertion (de Graaf et al., 2021). The assessment of global longitudinal strain must be considered, as a study observing systolic and diastolic function during follow‐ups in those with and without cardiac injury during hospitalization found no differences in the groups, but they did not utilize speckle tracking echocardiography (Catena et al., 2021). As such, subtle LV impairments may have gone unnoticed.

On the note of subacute cardiac changes, not all impairments are detectable by echocardiography. Using cardiac positron emission tomography, evidence of myocardial fatigue in PACS patients was found, implying the potential for subacute/chronic myocarditis.(Saricam et al., 2021) While LV subclinical dysfunction is present in those who have recovered from COVID‐19, these are not consistently noted with symptoms, and thus, the cardiac damage can be silent. Indeed, these findings from the literature do imply that even in the absence of cardiac symptoms, cardiac follow‐up with speckle tracking echocardiography can inform the clinician of cardiac impairments which may otherwise go undocumented. Should evidence of myocardial injury or acute coronary syndrome be present in the acute phase of infection, the 2–6‐month follow‐up must include a focus on cardiac monitoring and potentially utilize speckle tracking and magnetic resonance imaging (MRI), depending on the specific case characteristics (Mitrani et al., 2020). The relevance and clinical implications of these subclinical findings warrant further research as, during the pandemic, resource allocation and limitations must be considered.

### 2.2 Diastolic function

Diastolic dysfunction refers to an abnormal filling pattern in the left ventricle, and in many cases, presents large increases in end‐diastolic pressure during ventricular filling. A prospective observational study evaluated cardiopulmonary damage in 145 patients with COVID‐19 at two different visiting periods: 60 days and 100 days after their confirmed diagnosis (Sonnweber et al., 2021). Even at the second visit, 41% of the patients still exhibited symptoms. Using transthoracic echocardiography, it was found that diastolic dysfunction was present in 60% of the patients at the 60‐day follow‐up period, and 55% of the patients at the 100‐day follow‐up, with pericardial effusion diminished at the second follow‐up (Sonnweber et al., 2021). Eighteen participants who suffered severe COVID‐19 with acute respiratory distress syndrome were assessed using echocardiography during hospitalization with diastolic dysfunction, and no systolic dysfunction, found in nine of these patients at a 6‐month follow‐up (Daher et al., 2021).

The E/e’ ratio is an estimate of left ventricular filling pressure and refers to the early mitral inflow velocity and mitral annular early diastolic velocity, thereby allowing it to be an assessor of diastolic evaluation (Park & Marwick, 2011). At 6‐month follow‐up, no differences in cardiac parameters in those who had a myocardial injury during index hospitalization with COVID‐19 were found at rest (Fayol et al., 2021). However, low‐level exercise exacerbated cardiac function in this myocardial injury group, including an increase in the average E/e’ ratio and systolic pulmonary artery pressure (Fayol et al., 2021). Therefore, research is available to suggest that following recovery from COVID‐19, there can be sustained diastolic impairments and much of the research shows that this occurs with the presence of symptoms.

### 2.3 Right ventricular (RV) function

In acute severe COVID‐19 cases, those with RV dysfunction and dilatation had a greater mortality rate (Paternoster et al., 2021), and parameters such as depressed tricuspid annular plane systolic excursion (TAPSE) are associated with in‐hospital mortality (Silverio et al., 2021). Considering that TAPSE is an indicator of global RV function (Ueti et al., 2002), it, therefore, follows that the assessment of RV function can provide great insight into the prolonged cardiac impairments caused by COVID‐19. One hundred and five patients treated for mild severity COVID‐19 compared with healthy controls 3 months postinjury showed elevated systolic pulmonary artery pressure and RV myocardial performance index (Akkaya et al., 2021). Furthermore, it reduced TAPSE and RV global and free wall longitudinal strain, emphasizing RV dysfunction (Akkaya et al., 2021). These patients also had decreased RV fractional area change in comparison to patients with no history of COVID‐19 (Akkaya et al., 2021). Therefore, subclinical RV dysfunction can be detected even for 3 months following nonsevere COVID‐19. Building on this, a prospective cohort study with 40 patients, with either prolonged or new symptoms, who completed transthoracic echocardiography during hospitalization and at approximately 4‐month follow‐up showed that among all cardiac parameters, only RV diameter was significantly reduced (van den Heuvel et al., 2021). One hundred and thirty‐three days post‐COVID‐19 recovery follow‐up and comparison to healthy controls showed impairments in the global longitudinal and free wall strain (Ozer, Govdeli, et al., 2021), suggestive of subclinical RV dysfunction. It is important to note that this reduction in strain was only observed when the control group was compared with the subset population who suffered from severe COVID‐19 pneumonia (Ozer, Govdeli, et al., 2021). Indeed, another prospective study has shown that patients who suffer from moderate to severe COVID‐19 with no comorbidities had elevated RV end‐diastolic and end‐systolic area, reduced fractional area change, global longitudinal strain, and free wall strain 30 days after discharge (Gunay et al., 2021).

The presence of subclinical RV dysfunction in patients with COVID‐19 is important to discuss as reductions in RV strain are associated with greater mortality (Li et al., 2020). RV strain does appear to be a common finding during follow‐ups, even in patients with no history of cardiovascular or lung disease, and no presence of heart failure symptoms (Nuzzi et al., 2021), indicating that there is potential for silent cardiac (myocyte) damage and hemodynamic instability post‐COVID‐19 (Pelà et al., 2021).

Besides the use of echocardiography, other modalities include cardiac magnetic resonance imaging (CMR) and CT coronary angiography. In comparison to healthy controls, patients hospitalized with COVID‐19 show reduced RV ejection fraction with elevated native T1 values and reduced myocardial manganese uptake by CMR, implying impaired myocardial function (Singh et al., 2022). Furthermore, compared to patients with cardiac comorbidities, hospitalized patients with COVID‐19 still display reduced RV ejection fraction irrespective of COVID‐19 disease severity, presence of myocardial injury, or ongoing symptoms (Singh et al., 2022). Comparably, when assessed at 2–3 months postdischarge, hospitalized patients with COVID‐19 still displayed elevated native cardiac T1 and late gadolinium enhancement in comparison with healthy controls, although these parameters returned to normal at the 6‐month follow‐up (Cassar et al., 2021). However, RV ejection fraction was increased at this 6‐month follow‐up in comparison with healthy controls (Cassar et al., 2021). Therefore, different modalities of assessment of cardiac function all imply that RV function is altered during the follow‐up in patients who were hospitalized with COVID‐19, although this is not always dependent on symptoms.

### 2.4 Inflammation

Biomarkers of inflammation and cardiac injuries during the acute phase of COVID‐19 have been shown through multiple studies to be associated with increased in‐hospital mortality (Santoso et al., 2021; Tian et al., 2020). Furthermore, worsening echocardiographic parameters, such as RV dysfunction, appear to have an association with biomarkers of inflammation and cardiac injuries, such as high‐sensitivity cardiac troponin I in patients with COVID‐19 and underlying cardiovascular disease (Li et al., 2021). Thirty‐three COVID‐19 patients who were hospitalized but did not require ventilation between 48 and 71 days found that while patients presented with symptoms at the follow‐up, there was no significant damage or deterioration found with the echocardiography results (Daher et al., 2020). This may help explain the normal levels for the blood biomarkers, including inflammatory and markers of cardiac damage, and normal pulmonary function (Daher et al., 2020).

N‐terminal pro natriuretic peptide (NT‐proBNP), serum ferritin, and D‐dimer levels are among the biomarkers elevated at 100 days postconfirmed diagnosis (Sonnweber et al., 2021). In patients with varying severity who were followed up between 47 and 59 days after hospitalization, levels of D‐dimer and C‐reactive protein remained high in those who were discharged with elevated biomarkers (Mandal et al., 2021). Persistent fatigue and breathlessness were common symptoms at the follow‐up (Mandal et al., 2021). Therefore, considering the clotting and inflammatory potential of D‐dimer and C‐reactive protein, limitations in blood flow to the tissue, or chronic inflammation can help to explain the sustained symptoms. Serum samples collected at 40–60 days postinfection revealed elevated mitochondrial proteins and stress‐associated biomarkers such as peroxiredoxin 3, indicative of a mitochondrial stress response (Doykov et al., 2020). This was a very important finding as it can imply that cellular metabolism is impaired in patients even when they have recovered from COVID‐19, potentially leading to a long‐lasting inflammatory state. Notably, elevated blood biomarkers are not always found in follow‐up COVID‐19 research, as shown by a study observing blood biomarkers at 6‐month follow‐up in patients with a variety of index hospitalization serveries (Wu et al., 2021). Therefore, the risk of myocarditis in patients who recovered from COVID‐19 is unknown, however, more research correlating the index hospitalization severity with follow‐up biomarker data can clarify this question.

Plasma samples collected from patients with sustained symptoms have shown the presence of inflammatory molecules containing amyloid deposits and are resistant to fibrinolysis in long and acute COVID‐19 patients in comparison with healthy individuals or those with type 2 diabetes (Pretorius et al., 2021). Furthermore, with the potential for sustained microclots in patients with PACS/long COVID symptoms (Pretorius et al., 2021), there may be limited blood flow to the tissues, thus starving them of oxygen and leading to altered cardiometabolic activity and muscle metabolism. Acute COVID‐19 severity is known to result in different cardiac complications, be it mild, moderate, and severe acute infection. Common findings initially included elevations of T1 values and T2 values, which are markers of inflammation and edema, respectively, along with abnormal late gadolinium enhancement patterns. However, these implications were not reproduced in many large‐scale follow‐up studies on patients who recovered from mild injury COVID‐19 (Satterfield et al., 2021). In moderate to severe acute infection, there is evidence of persistent ventricular dysfunction, and more severe COVID‐19 infection as indicated by elevated C‐reactive protein or D‐dimer also shows greatly elevated T2 (edema) values (Pan et al., 2021; Satterfield et al., 2021). Therefore,the severity of index hospitalization must be considered in research observing systolic, diastolic, RV, and inflammatory changes in COVID‐19 follow‐ups.

### 2.5 Complications with therapeutics approaches

Sustained symptoms in patients recovered from COVID‐19 appear to mimic a chronic fatigue state, with thoughts that the disease may eventually lead to chronic fatigue syndrome (Komaroff & Bateman, 2020). Exercise therapy must be considered with precaution as altered metabolic capabilities such as those in chronic fatigue syndrome can limit their physical activity potential (Patrick Neary et al., 2008). Current research implies that oxygen saturation, heart rate, blood pressure, and symptomatology with an emphasis on dyspnea and rate of perceived exertion must be monitored during exercise, with unique, individually tailored programs required for the greatest therapeutic return (Calabrese et al., 2021). Considering the lack of information surrounding pharmaceutical therapy in symptomatic patients who recovered from COVID‐19, carefully monitored exercise provides a useful tool.

Reduced exercise capacity in those discharged from the hospital following COVID‐19 appears to be common, with 246 from 1250 survivors showing some reduced activity capacity (Chopra et al., 2021), which can occur irrespective of common postviral complications or sustained symptoms (Lam et al., 2021). Recently, an exercise study included a comparison of discharged COVID‐19 patients with self‐reported reduced exercise capacity to discharged patients with normal exercise capacity and controls (Brown et al., 2022). When indexed to body surface area, those with a history of COVID‐19 showed a reduced indexed left ventricular end‐systolic volume and elevated LVEF. Furthermore, those with reduced exercise capacity also had reduced indexed oxygen consumption, indexed stroke volume, and indexed left ventricular end‐diastolic volume. This study therefore suggests that hemodynamic impairments may occur resulting from a failure to augment stroke volume and cardiac output during exercise. Considering that systolic or diastolic function was not abnormal, the authors suggested that inadequate preload may be causing the impairments in stroke volume. Specifically, blood volume is reduced in postviral chronic fatigue syndrome (conditions similar to COVID‐19 with sustained symptoms) (Natelson et al., 2021), which can reduce ventricular filling during exercise.

**FIGURE 1:**
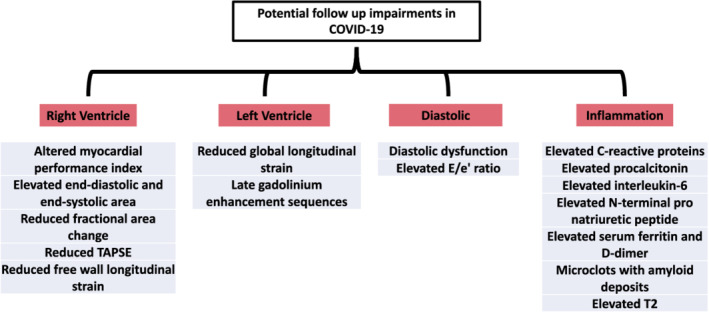
Clinical follow‐up complications arising from severe acute COVID‐19. Parameters shown to be impaired in follow‐up research on patients recovered from COVID‐19.

To synthesize available literature, a recent systematic review of cardiovascular sequelae after COVID‐19 recovery evaluated 35 studies with a median follow‐up time of 48 days. This review found that inflammatory signs on magnetic resonance imaging, pericardial effusion, and late gadolinium enhancement are common acute findings before 3 months (Ramadan et al., 2021). Reduced left ventricular global longitudinal strain, late gadolinium enhancement, and diastolic dysfunction are often found at 3‐to‐6‐month follow‐up (Ramadan et al., 2021). The sustained cardiac damage can be due to the initial elevated inflammatory responses, and further result in a heightened risk of adverse cardiac events. Indeed, COVID‐19 patients are more likely to develop heart failure, heart attack, and arrhythmias (Ramadan et al., 2021). However, it is important to note that not all studies show similar cardiac involvement following COVID‐19, thus suggesting that there may be a need for a personalized approach to the assessment of these patients. Furthermore, not all patients in the follow‐up studies exhibit symptoms of COVID‐19, implying that not all the results presented in these studies are from PACS/long COVID patients. Figure 1 is included to summarize the potential cardiac impairments which can be found during follow‐up in those with severe acute COVID‐19.

### 2.6 Proof of concept presentation

Prior to testing, this study was approved by the University of Regina Research Ethics Board (REB#2020‐073), and the participants signed a written informed consent form. Participants completed a medical history form which included general questions related to their physical activity level, medications, caffeine and alcohol consumption, sleep, and history of mild traumatic brain injury. Participants were all at least 18 years old. All participants were unvaccinated at the time of data collection. Participants did not exercise for at least 8 h and did not intake any alcohol at least 24 h before testing. A noninvasive cardiac sensor (LLA Recordis™; LLA Technologies) was used to record the cardiac cycle timing and force production parameters of the heart (Singh et al., 2021). The sensor was snapped into a single adhesive ECG gel‐electrode and placed approximately 1 cm above the xiphoid process on the sternum of the chest over the skin. Participants rested in a supine position for at least 1‐min prior to data collection and during data collection. For participants with cardiac and respiratory disease, this procedure was completed under researcher supervision during their clinic visit. For the COV‐SS participants, the device was delivered to them and the recordings were completed twice per week by the participants following instructions from the researcher. The average of their recordings was taken. These recordings were taken twice per week and at the same time of day for each recording. For the acutely infected participants, the device was delivered to their doorstep and the participants were guided on device placement during a subsequent telephone call by the same researcher. Participants collected data acutely and were asked to follow up thereafter.

While in the supine position, the sensor was placed on the participant's sternum. The sensor was turned on for 1 min and recorded the cardiac vibrations, thus allowing the mapping of the cardiac cycle (Singh et al., 2021). The analysis of the cardiac signal collected at the sternum has been reported elsewhere (Singh et al., 2021). Data were preprocessed with a first‐order Butterworth bandpass filter with a low cutoff frequency of 1 Hz and a high cutoff frequency of 30 Hz filter. Following preprocessing, an in‐house, independent (proprietary) algorithm (LLA Technologies Inc.) was applied to identify the fiducial point of the cardiac cycle. These included the following: mitral valve closure (MVC), aortic valve opening (AVO), aortic twist (ATT), aortic systole, REP, aortic valve closure (AVC), ventricular untwisting, and mitral valve opening (MVO). Following the analyses of the fiducial points, temporal features being heart rate, diastole (mitral valve closure—mitral valve open time), systole (aortic valve open—aortic valve closure time), IVCT (mitral valve closure—aortic valve open), IVRT (aortic valve closure—mitral valve open time), mitral valve open to E wave (MVO to E), and end of rapid ejection (REP). The magnitude of the cardiac contraction at the sternum was calculated in milligravity (mG) as twist force (TF). This is done by quantifying the vibrational activity produced by ventricular contraction and picked up by the accelerometer at the sternum.

Shapiro Wilk tests for normality suggested that not all data parameters were normally distributed (*p* < 0.05). However, as normality tests such as the Shapiro Wilk reduce in power with smaller sample sizes, a conservative approach was taken to utilize nonparametric analyses for all data parameters (Mohd Razali & Yap, 2011). Kruskal–Wallis one‐way ANOVAs were used to compare the four independent groups for each dependent variable. If statistical significance was found, pairwise comparison using Dunn's test and the p value was adjusted using the Benjamini‐Hochberg technique with *p* < 0.05 being considered as significant (Dinno, 2015). Wilcoxon signed‐rank tests were performed to analyze the pre‐ and postresponses for the COV‐A and their follow‐up assessment (COV‐F). Performance indices were calculated according to the literature (Biering‐Sorensen et al., 2015; Goroshi & Chand, 2016):

- Diastolic performance index: DPI = IVRT/ejection time
- Systolic performance index: SPI = IVCT/ejection time
- Heart (often referred to as Tei index or myocardial index) performance index: HPI = (IVCT+IVRT)/Ejection time

Normal values for those with no history of cardiac disease for the cardiac cycle, and timing intervals (milliseconds, ms) vary based on both age and sex. More specifically, research has shown that from the age groups of 20–39 to those who are 60 and above, IVRT ranges from 78 to 106 ms for females and 78 to 109 ms for males, while IVCT ranges from 32 to 38 ms for females and 34 to 36 ms for males (Biering‐Sorensen et al., 2016). The systolic or ejection time ranges from 291 to 289 ms for females and 284 to 279 ms for males, thus the IVCT and IVRT changes contribute to the elevation in HPI from 0.38 to 0.51 in females and 0.40 to 0.53 in males (Biering‐Sorensen et al., 2016). Detailed further in the discussion, those with a history of major adverse cardiac events often see the alteration in these cardiac cycle parameters. The HPI is well studied and is considered to be reflective of global myocardial performance, as it includes both the systolic and diastolic components of the cardiac cycle, and the reproducibility with different echocardiographic modalities, including M‐mode and pulsed‐wave Doppler has been shown (Biering‐Sorensen et al., 2016; Gaibazzi et al., 2005).

### 2.7 Results

Fourteen participants with respiratory complications of either COPD and/or asthma, 18 participants with cardiac complications, 17 COV‐SS, and 23 COV‐A participants were enrolled in this study. The participant demographics are presented in Table 1. No participants were vaccinated at the initial time of infection. Many participants did have multiple cardiac or respiratory disease prevalence.

**TABLE 1:** Demographic information of the four groups

	Respiratory	Cardiac	COV‐SS	COV‐A
Sample size (*n*)	14	18	17	23
Age (years) ± SD	47 ± 17	60 ± 12	48 ± 14	43 ± 18
Height (cm) ± SD	169 ± 8	175 ± 10	170 ± 11	174 ± 9
Body mass (kg) ± SD	86 ± 18	97 ± 22	84 ± 22	79 ± 16
Female (%)	28	17	52	39
Disease				
% COPD	50	—	4	4
%Asthma	64	—	—	—
%Hypertension	—	72	—	—
%Diabetic	—	44	—	—
%Include other cardiac disease	—	83	9	4
Symptoms^a^				
%General pain	7	6	—	48
%Chest tightness	21	11	18	4
%Dyspnea	7	6	29	26
%Fatigue	7	28	100	74
Variant				
%L‐type	—	—	35	35
%B.1.617.2	—	—	65	65

Abbreviations: COV‐SS, COVID‐19 with sustained symptoms; COV‐A, acute COVID‐19; COPD, Chronic obstructive pulmonary disease.

^a^ All COV‐SS participants showed variations in fever and all COV‐A presented with at least mild fatigue.

#### 2.7.1 Systolic and isovolumic time

Systolic time was significantly different (*H* = 9.52, *p* = 0.02), with a median systolic time of 268 ms for the respiratory group, 284 ms for the cardiac group, 324 ms for the COV‐SS group, and 299 ms for the COV‐A group. Pairwise analysis revealed that systolic time in COV‐SS participants was significantly greater than in those with respiratory disease (*p* < 0.01). IVCT was significantly different (H = 12.22, *p* < 0.01), with a median of 34 ms for the respiratory group, 38 ms for the cardiac group, 32 ms for the COV‐SS group, and 37 ms for the COV‐A group. Pairwise analysis revealed that IVCT in COV‐SS participants was lower than in participants with cardiac disease (*p* < 0.01) and the COV‐A group (*p* < 0.05) (Table 2).

**TABLE 2:** Kruskal–Wallis one‐way ANOVA results (Median ± SD) for each group

	Respiratory (*n* = 14)	Cardiac (*n* = 18)	COVID‐19 Sustained Symptoms (*n* = 17)	Acute COVID‐19 (*n* = 23)	H‐Stat	Kruskal–Wallis	Dunn Comparison
Heart Rate (bpm)	79 ± 12	65 ± 17	69 ± 10	62 ± 17	8.38	<0.05	<0.05^a^
Systolic Time (ms)	268 ± 30	284 ± 56	324 ± 44	291 ± 60	9.52	<0.05	<0.05^b^
Diastolic Time (ms)	360 ± 125	489 ± 158	422 ± 111	543 ± 194	5.79	0.12	N.S.
IVCT (ms)	34 ± 8	38 ± 5	32 ± 4	36 ± 7	12.22	<0.01	<0.01^c^ <0.05^d^
IVRT (ms)	89 ± 15	88 ± 11	88 ± 8	95 ± 13	3.32	0.35	N.S.
REP (ms)	64 ± 15	74 ± 15	78 ± 12	80 ± 27	6.18	0.10	N.S.
MVO_E (ms)	45 ± 9	48 ± 9	46 ± 7	47 ± 8	0.98	0.81	N.S.
Twist Force (mG)	16 ± 9	14 ± 6	12 ± 4	17 ± 6	7.82	0.05	N.S.
HPI	0.45 ± 0.11	0.45 ± 0.12	0.38 ± 0.04	0.44 ± 0.09	14.13	<0.01	<0.05^b^ <0.01^c^ <0.01^d^
SPI	0.12 ± 0.04	0.13 ± 0.04	0.10 ± 0.02	0.13 ± 0.04	16.76	<0.001	<0.05^b^ <0.01^c^ <0.01^d^
DPI	0.33 ± 0.07	0.30 ± 0.09	0.28 ± 0.03	0.32 ± 0.08	8.78	<0.05	<0.05^b^ <0.05^c^ <0.05^d^

Abbreviations: DPI, Diastolic performance index; HPI, Heart performance index; IVCT, Isovolumic contraction time; IVRT, Isovolumic relaxation time; MVO_E, Mitral valve open to E wave; REP, Rapid ejection period; SPI, Systolic performance index.

^a^ COV‐A vs Respiratory.

^b^ COV‐SS vs respiratory.

^c^ COV‐SS vs cardiac.

^d^ COV‐SS vs COV‐A.

#### 2.7.2 Performance indices

HPI was significantly different (*H* = 14.13, *p* < 0.01), with a median of 0.45 for the respiratory group, 0.45 for the cardiac group, 0.38 for the COV‐SS participants, and 0.44 for the COV‐A group. Pairwise analysis revealed that HPI in COV‐SS participants was lower than in those with respiratory disease (*p* < 0.01), cardiac disease (*p* < 0.01), and COV‐A (*p* < 0.01). DPI was significantly different (*H* = 8.37, *p* < 0.05), with a median of 0.30 for the respiratory group, 0.30 for the cardiac group, 0.28 for the COV‐SS participants, and 0.32 for the COV‐A group. Pairwise analysis revealed that DPI in COV‐SS participants was lower than in those with respiratory disease (*p* < 0.05), cardiac disease (*p* < 0.05), and COV‐A (*p* < 0.05). SPI was significantly different (*H* = 16.76, *p* < 0.001), with a median of 0.13 for the respiratory group, 0.12 for the cardiac group, 0.10 for the COV‐SS participants, and 0.13 for the COV‐A group. Pairwise analysis revealed that SPI in COV‐SS participants was lower than in those with respiratory disease (*p* < 0.05), cardiac disease (*p* < 0.001), and COV‐A (*p* < 0.01) (Table 2).

#### 2.7.3 Acute and follow‐up comparison

The mean assessment day postsymptom onset or positive test result was 5 ± 4 in the COV‐A group and 38 ± 16 in the COV‐F group. There was a significant increase in HPI in the COV‐A group compared with the COV‐F group (*Z* = −2.01, *p* < 0.05). No other values were significantly different (Table 3).

**TABLE 3:** Wilcoxon signed‐rank test results (Median ± SD)

Parameter	Acute COVID‐19	Follow‐up COVID‐19	Significance
Heart Rate (bpm)	62 ± 17	65 ± 11	NS
Systolic Time (ms)	291 ± 60	306 ± 34	NS
Diastolic Time (ms)	543 ± 194	481 ± 156	NS
IVCT (ms)	36 ± 7	36 ± 7	NS
IVRT (ms)	95 ± 13	94 ± 14	NS
REP (ms)	80 ± 27	80 ± 17	NS
MVO_E (ms)	47 ± 8	48 ± 7	NS
Twist Force (mG)	17 ± 6	14 ± 5	NS
HPI	0.44 ± 0.09	0.43 ± 0.06	<0.05
SPI	0.13 ± 0.04	0.11 ± 0.03	NS
DPI	0.32 ± 0.08	0.30 ± 0.04	NS

Abbreviations: DPI, diastolic performance index; HPI, Heart performance index; IVCT, Isovolumic contraction time; IVRT, Isovolumic relaxation time; MVO_E, Mitral valve open to E wave; NS, nonsignificant; REP, Rapid ejection period; SPI, Systolic performance index.

## 3 CONCLUSION

Depressed global longitudinal strain, subclinical RV dysfunction, diastolic dysfunction, and elevated inflammatory biomarkers appear to be common in PACS/long COVID. Given the cardiac damage which can occur in mild to severe acute COVID‐19, in combination with delays in access to healthcare, systolic dysfunction, and clinical heart failure are likely to be more prominent.(Satterfield et al., 2021) Observing cardiopulmonary and multisystem symptoms, in combination with physiological and structural cardiac abnormalities, can help to reduce the progression of cardiac dysfunction (Satterfield et al., 2021). More recent research demonstrates that these impairments can last for months postinfection. Indeed, while symptoms may be well known, the percentage of individuals who suffer from long‐term symptoms varies from study to study. Whether these studies relate to cardiac symptoms or complications is not understood, and the exact methods for cardiac follow‐ups (echocardiography, CMR, etc.) are still under investigation. Limited research is also available to show that elevated blood biomarkers can remain for months following the acute infection period, however, more research is required to elucidate the physiological mechanisms.

The proof‐of‐concept reports for the first time in the literature the unique cardiac dysfunction of significantly elevated systolic time in COV‐SS. Furthermore, the systolic and isovolumic times influence the performance indices, which are all reduced in COV‐SS in comparison with all other groups. Speculation as to why these changes may occur include cardiomyocyte fatigue, considering that systolic timing event was significantly increased and IVCT was significantly decreased (Table 2). Cardiomyocytes are densely populated with mitochondria, thus allowing the myocardium to sustain years of repeated contraction. There is therefore potential that these findings imply a mitochondrial stress response in these patients and may be indicative of excessive fatigue and impaired cellular metabolism, requiring an upregulated mitochondrial response (Doykov et al., 2020). This fatigue helps to explain why cardiac contraction in COV‐SS is sustained in comparison with the other groups. Other complications can be related to the failure for augmentation in stroke volume in symptomatic COVID‐19 patients, which can impact LV preload. In doing so, this can result in reduced blood volume, and by the Frank‐Starling mechanism, theoretically result in reduced contractility. However, we did not observe altered twist force in our participants, thus, if preload is reduced, and contractility is sustained, in order to force ejection, there can be a sustained contraction or systolic period. Limitations of this study include the small and uneven sample sizes in each group. Larger sample sizes can help to minimize type 2 error rates. Larger sample sizes can also help to stratify groups in greater detail, including better control for the presence of comorbidities and multiple cardiac and respiratory diseases. Finally, external variables such as the uneven age distribution, blood pressure, and fitness levels of the participants may influence the cardiac cycle times and the performance indices.

## AUTHOR CONTRIBUTIONs

Jyotpal Singh, Lanishen Bhagaloo, and J. Patrick Neary conceived and designed the experiments, collected the data, and contributed to the writing and revising of the manuscript. Eric Sy, Andrea J. Lavoie, Payam Dehghani, Holly A. Bardutz, Cameron S. Mang, and Josef Buttigieg contributed to the understanding and analyses of the data and the revision of the manuscript. All authors have seen and approved the final manuscript.

## FUNDING INFORMATION

The authors thank LLA Technologies Inc. and Mitacs Inc. for supporting this research through the Mitacs Accelerate Program (MITACS ACCELERATE IT25895).

## CONFLICT OF INTEREST

The authors have no conflict of interest in this project.
